# Growth dynamics of brain metastases differentiate radiation necrosis from recurrence

**DOI:** 10.1093/noajnl/vdac179

**Published:** 2022-12-08

**Authors:** Beatriz Ocaña-Tienda, Julián Pérez-Beteta, David Molina-García, Beatriz Asenjo, Ana Ortiz de Mendivil, David Albillo, Luís A Pérez-Romasanta, Elisabeth González del Portillo, Manuel Llorente, Natalia Carballo, Estanislao Arana, Víctor M Pérez-García

**Affiliations:** Mathematical Oncology Laboratory, University of Castilla-La Mancha, Ciudad Real, Spain; Mathematical Oncology Laboratory, University of Castilla-La Mancha, Ciudad Real, Spain; Mathematical Oncology Laboratory, University of Castilla-La Mancha, Ciudad Real, Spain; Department of Radiology, Hospital Regional Universitario Carlos Haya, Málaga, Spain; Department of Radiology, Sanchinarro University Hospital, HM Hospitales, Madrid, Spain; Radiology Unit, MD Anderson Cancer Center, Madrid, Spain; Radiation Oncology Service, Salamanca University Hospital, Salamanca, Spain; Radiation Oncology Service, Salamanca University Hospital, Salamanca, Spain; Radiology Unit, MD Anderson Cancer Center, Madrid, Spain; Radiology Unit, MD Anderson Cancer Center, Madrid, Spain; Department of Radiology, Fundación Instituto Valenciano de Oncología, Valencia, Spain; Mathematical Oncology Laboratory, University of Castilla-La Mancha, Ciudad Real, Spain

**Keywords:** Brain metastases, mathematical oncology, radiation necrosis, stereotactic radiosurgery

## Abstract

**Background:**

Radiation necrosis (RN) is a frequent adverse event after fractionated stereotactic radiotherapy (FSRT) or single-session stereotactic radiosurgery (SRS) treatment of brain metastases (BMs). It is difficult to distinguish RN from progressive disease (PD) due to their similarities in the magnetic resonance images. Previous theoretical studies have hypothesized that RN could have faster, although transient, growth dynamics after FSRT/SRS, but no study has proven that hypothesis using patient data. Thus, we hypothesized that lesion size time dynamics obtained from growth laws fitted with data from sequential volumetric measurements on magnetic resonance images may help in discriminating recurrent BMs from RN events.

**Methods:**

A total of 101 BMs from different institutions, growing after FSRT/SRS (60 PDs and 41 RNs) in 86 patients, displaying growth for at least 3 consecutive MRI follow-ups were selected for the study from a database of 1031 BMs. The 3 parameters of the Von Bertalanffy growth law were determined for each BM and used to discriminate statistically PDs from RNs.

**Results:**

Growth exponents in patients with RNs were found to be substantially larger than those of PD, due to the faster, although transient, dynamics of inflammatory processes. Statistically significant differences (*P* < .001) were found between both groups. The receiver operating characteristic curve (AUC = 0.76) supported the ability of the growth law exponent to classify the events.

**Conclusions:**

Growth law exponents obtained from sequential longitudinal magnetic resonance images after FSRT/SRS can be used as a complementary tool in the differential diagnosis between RN and PD.

**Key Points**

1. Growth laws exponents allow differentiating radiation necrosis from progressive disease.
2. Volumes measured from MR images can be used for the identification of radiation necrosis in BMs.

**Importance of the Study**

Discriminating radiation necrosis (RN) events from recurrences after fractionated stereotactic radiotherapy or single-session stereotactic radiosurgery is a major point in the management of brain metastases (BMs). An erroneous diagnosis could lead to either unnecessary invasive actions and counterproductive treatments when RN is confused with recurrences or delays in treatments in the opposite situation. Conventional images have been thought not to be appropriate to discern between both conditions since they show similar characteristics on magnetic resonance images. Our study provides simple ways to differentiate RN from PD based on the study of the growth dynamics of BMs, assessed from the volume of the lesions measured on T1-weighted MR images.

Brain metastases (BMs) are the most common intracranial tumor, where 10%–40% of patients who die from cancer suffered from BMs.^1,2^ Some primary cancers such as lung (non-small cell lung cancer and small cell lung cancer), melanoma, and breast cancer^2^ are more likely to metastatize in the brain. Advances in the treatment of these primary tumors have led to longer patient survival, and thus to an increase in the incidence of BMs.^3^

Stereotactic radiation therapy can be delivered as either multiple fractions (fractionated stereotactic radiotherapy—FSRT—) or as a single fraction of high-dose treatment (stereotactic radiosurgery—SRS—, generally 18–24 Gy),^4^ due to its excellent local control rates, especially in lesions below 2 cm in diameter.^5^ Radiation necrosis (RN) is a challenging complication after SRS, since it can mimic true progression not only in appearance but also in clinical symptoms.^6^ It occurs in up to 25% of patients after SRS.^7^ Also, the coexistence of tumor and RN further complicates diagnosis.

The gold-standard diagnostic test for differentiating tumor progression from RN is biopsy sampling,^4^ but there are limitations in its use. Conventional magnetic resonance imaging (MRI) is not appropriate to discern between RN and PD, since both show similar characteristics. In this sense, other methods have been used to differentiate both entities, including perfusion MR imaging,^8^ positron emission tomography,^9^ the use of artificial intelligence (AI)^10^ and radiomic methods,^11,12^ or changes in the shape of anatomical boundaries.^13^ However, many of those methods have not been validated by independent groups and additional studies are required, or they are based on complex computational methodologies. There is a need for diagnostic techniques based on routinely used imaging methods, such as post-contrast T1-weighted images (T1–WI).^13^

In this work, our goal was to study whether growth laws, which have been shown to effectively describe the longitudinal growth dynamics of human tumors,^14^ could discriminate between PD or RN after SRS.

## Materials and Methods

### Patients

Patients included were all participants in a retrospective, multicenter, nonrandomized study, approved by the institutional review boards of 6 hospitals. All patients were diagnosed with one or several BMs in the period 2007–2021 and followed up with MRI according to the standard clinical practice.^15^ The eligibility criteria for the study were as follows: (1) Having received SRS or FSRT at any time during the course of the disease without concurrent whole brain radiotherapy (WBRT). BMs with WBRT at least one month before SRS or RSRT were included., (2) A minimum of 3 consecutive imaging studies, including volumetric contrast-enhanced (CE) T1-weighted MRI sequences (slice thickness ≤2.00 mm) with no substantial imaging artifacts, at different time points after SRS without additional treatment., (3) An increase in tumor volume at each of the 3-time points., (4) Patients with prior surgical resection of the metastasis were excluded to avoid confounding effects., and (5) Patients were included regardless of their systemic therapy.

A total of 101 BMs from 86 patients met the inclusion criteria after a thorough revision of the 1133 BMs in the full dataset. Of these, 59% (*n* = 60) underwent PD and the others (*n* = 41) developed RN. A summary of patient characteristics is provided in Table 1.

**Table 1.** Summary of Patient Characteristics

	n (%)
Sex	
Male	44 (51.2)
Female	42 (48.8)
Age	
Median (range)	55 (33–78)
Primary tumor histology	
NSCLC	57 (66.3)
Breast	11 (12.8)
** SCLC**	7 (8.1)
** Melanoma**	2 (2.3)
** Others**	9 (10.5)
Number of metastases at diagnosis	
** 1**	33 (38.4)
** 2**	22 (25.6)
** 3**	10 (11.6)
** ≥ 4**	21 (24.4)
Systemic therapy	
** No**	44 (51.2)
** Yes (See a list in** Supplementary Table S1)	42 (48.8)
Radiation therapy	
** Single-session stereotactic radiosurgery**	62 (61.4)
** Dose (Gy) (median [range])**	19.75 (15–24)
** Fractionated stereotactic radiotherapy**	39 (38.6)
** Number of fractions (median [range])**	5 (3–10)
** Dose per fraction (Gy) (median [range])**	6.2 (3.7–16)
** Upfront whole brain radiotherapy**	20 (19.8)

### Radiation Treatments and RN Diagnosis

All BMs in the study were treated with either FSRT or SRS. Only a fraction of them (20/101) had previously received whole brain radiotherapy (WBRT) for the BMs. The median time between the end of WBRT and SRS was 6.4 months (1.1–23.4 months).

The diagnosis of the RN was based on the analysis of the surgical specimen. When surgery was performed, at least 50% necrosis and/or inflammation was considered as RN. When surgery was not performed, the diagnosis was based on the following criteria: (1) increased T1 contrast enhancement located in the irradiated area with central hypointensity and increased peripheral edema, (2) substantial regression or stability (for ≤4 months) of enhancing areas on serial follow-up MRI scans without additional treatment or after bevacizumab, and (3) reduced relative cerebral blood volume (rCBV) and F-18 fluorodeoxyglucose (FDG) in the absence of any highly vascularized nodular area within the CE MR images of the lesion.^16^

### Imaging and Follow-up

Patients were followed up with a volumetric MRI scan and clinical follow-up appointments. The typical time spacing between MRI studies was about 3 months for the institutions participating in the study. In our dataset, the median time between the first 2 MRI studies was 3.04 months, while for the second it was 2.42 months.

The volumetric CE T1-WI on MR imaging used to delineate the BMs and compute their volumes was gradient echo, using either 3D spoiled gradient-recalled echo or 3D fast-field echo after intravenous administration of a single dose of gadobenate dimeglumine (0.10 mmol/kg) with a 6-to 8-minute delay. MR studies were performed in the axial or sagittal plane with a 1.0 T (*n* = 4), 1.5 T (*n* = 278), or 3.0 T (*n* = 21) MR imaging unit, where n represents the number of MR scans used in this work. Imaging parameters were slice thickness of 0.5–2.0 mm (median 1.3 mm) and 0.4–1.0 mm (median 0.5 mm) pixel spacing.

### Tumor Segmentation

T1-weighted images were retrospectively analyzed by the same image expert and reviewed by both an image expert with more than 6 years of expertise in tumor segmentation and a senior radiologist with 27 years of experience. Segmentations were performed by importing the DICOM files into the scientific software package MATLAB (R2022a, The MathWorks, Inc., Natick, MA, USA). Each BM lesion was automatically delineated using a gray-level threshold chosen to identify the CE tumor volume. Segmentations were then corrected manually, slice by slice, as described previously.^17^ However, any segmentation process based on threshold selection and manual correction could be performed using other open-source software such as 3DSlicer. Necrotic tissue was defined as hypointense tumor regions inside CE tissue. CE and necrotic areas of the lesions were reconstructed, and the tumor interfaces were rendered in 3D. Tumor volume was computed as the volume within the surface delimiting CE areas.

### Longitudinal Growth Analysis: Scaling Laws

The so-called Von Bertalanffy growth model^18^ describes longitudinal tumor growth as a function of the volume of the lesion. We focus on growing tumors for which the tumor cell loss is negligible so that growth is governed by the differential equation.


$$\begin{array}{l} \frac{dV}{dt}=\alpha V^{\beta },\;\;\;\;\;\;\;\;\;V\left(t_{0}\right)=V_{0} \end{array}$$ (1)

This equation has recently been shown to describe the dynamics of human BM growth.^18^ Since our dataset includes patients with 3 consecutive longitudinal measurements post-SRS without additional treatments, the data (*V*_0_*, V*_1_*, V*_2_) allow us to identify the 3 relevant model parameters for each lesion (*V*_0_*,α,β*). The Von Bertalanffy equation is a mathematical growth model. Thus, it is necessary to have volumes that increase over time, for it to be used to obtain the growth exponent.

### Two Time-Point Analysis

The calculation of the growth exponent *β* requires 3 consecutive MR images with a volume increase. However, often only 2 follow-ups are available in clinical practice. For each pair of volumetric measurements (*V*_0_*, V*_1_) in consecutive time points (*t*_0_*, t*_1_) in longitudinal studies, we can obtain the instantaneous tumor growth rates using the equations.


$$\begin{array}{l} \lambda_{1}=\frac{1}{\left(t_{1}-t_{0}\right)}log\frac{V_{1}}{V_{0}},\;\;\;\;\lambda_{2}=\frac{1}{\left(t_{2}-t_{1}\right)}log\frac{V_{2}}{V_{1}}, \end{array}$$ (2)

The exponent *λ* was computed twice for each BM with 3 consecutive longitudinal measurements as described in Equation (2). First for $\lambda_{1}$, taking into account the first and second available points; and secondly, to compute $\lambda_{2}$, using data from the second and third-time points.

### Sensitivity Analysis

Errors in tumor segmentation volumes (*V*_0_*, V*_1_*, V*_2_) may have an effect on the estimated parameters, and more specifically, on the growth exponent (*β*). To ensure that small variations in the computed volume did not influence our results, a sensitivity analysis was performed on the model (2). A random error smaller or equal to ±5% was added to every volume and repeated for each BM 200 times. The average of the 200 computed β* was constrained to have a difference smaller than 0.5 when compared with the *β*obtained from the measured volumes, that is to say, $\left|{*}_{av}-\right|<0.5$. We performed the same calculation but required the median value of the exponent β*.

### Statistical Analysis

Statistical analyses were performed using MATLAB 2022a (Mathworks) and SPSS (Statistical Package for the Social Sciences, v25.00 IBM) software. The normality of the variables was assessed via the Kolmogorov–Smirnov test. The Kruskal–Wallis test was conducted with adjustment for multiple comparisons, to determine statistically significant differences for non-parametric data (the scaling law growth factor,*β*). *P*-values smaller than .05 were considered to be statistically significant. A receiver operating characteristic curve was built in SPSS calculating the area under the curve (AUC) as a measure of accuracy to establish how good a BM classification would be.

### Ethical Approval

We have complied with all relevant ethical regulations. Human data were obtained in the framework of the study MetMath (Metastasis and Mathematics), a retrospective, multicenter, nonrandomized study approved by the corresponding institutional review boards.

## Results

**Figure 1.**
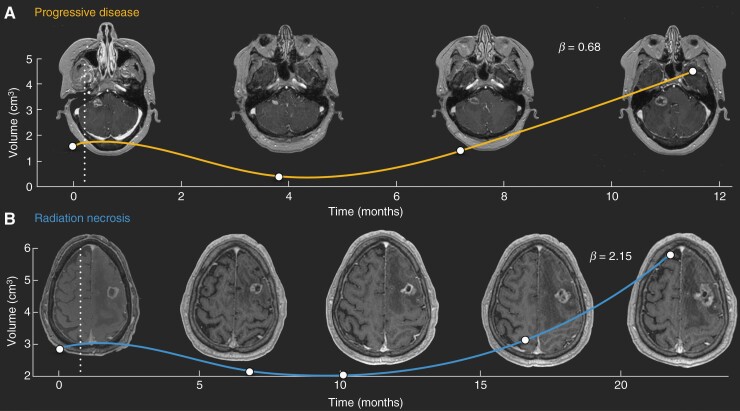
Longitudinal dynamics were observed in (A) Lesion with progressive disease (PD) post-SRS and (B) an irradiated BM diagnosed with radiation necrosis (RN). SRS treatment times are marked with a vertical dashed line. White dots are the measured volumes, and the solid lines are the result of interpolating longitudinal volumetric data (shown only to guide the eye). Axial slices of the contrast-enhanced (CE) T1-weighted magnetic resonance imaging (MRI) sequences are displayed.

The growth dynamics of recurrent BMs post-SRS and RN events were studied. Tumor recurrence may require prompt therapeutic action, typically in the form of brain surgery or re-irradiation when appropriate (an example is shown in Figure 1A). However, RN events lead also to lesion increase which usually regresses spontaneously, requiring no further therapeutic action (Figure 1B).

**Figure 2.**
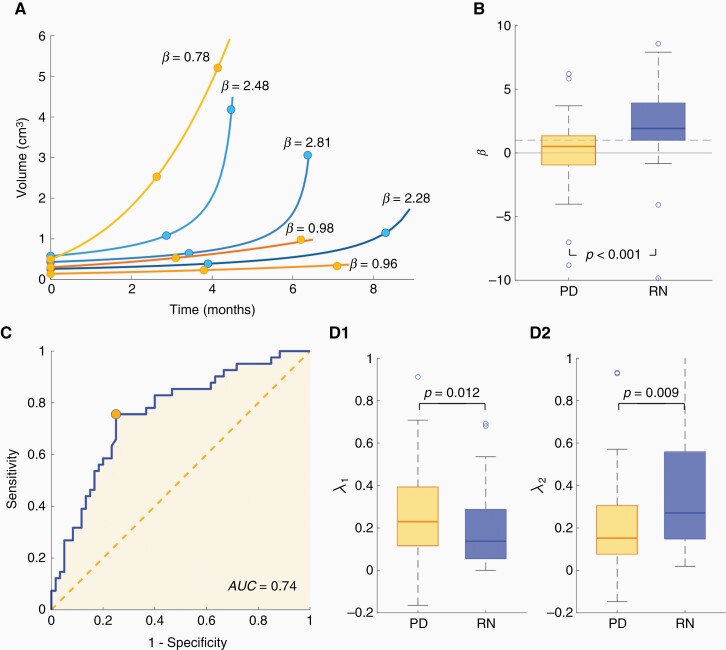
(A) Fitting curves and the growth exponents obtained from Equation (1) for a number of brain metastases (BMs), where dots correspond to the volumes measured. (B) Box plots comparing the growth exponents *β* obtained for post-SRS recurrence of BM (PD, *n* = 60) vs radiation necrosis (RN) events (*n* = 41). (C) receiver operating characteristic curve for the distinction between recurrent BM post-SRS and RN. (D) Box plots showing the growth rates λ obtained for the same subgroups by an exponential fit Equation (2) choosing (A**1)** the first 2 time points (λ _1_) and (D2) the last 2 time points (λ _2_).

The differences between the growth exponents obtained from fitting longitudinal data for the groups of PD and RN were then studied. The growing segment of the curve was first fitted to Equation (1) as described in “Methods” (Figure 2A). The mean value of the growth exponent for the 60 BMs with PD after SRS was found to be *β* = 0.52, while for the 41 RN events included in the study it was *β* = 2.10. The Kruskal-Wallis test for the comparison between the RN and the PD showed significant differences (*P* < .001) between the 2 groups. The box plot in Figure 2B shows this comparison. Thus, RN events displayed large growth exponents, i.e. fast growth acceleration, faster indeed than those of BM recurrence post-SRS.

The area under the receiver operating characteristic curve in Figure 2C illustrates the ability of the exponent *β* obtained from longitudinal growth data to classify events either as RN events or recurrences. The result obtained was AUC = 0.740, with 0.756 sensitivity and 0.750 specificities. Based on the mathematical understanding of tumor growth dynamics, this metric may, therefore, complement other diagnostic tests in differentiating RN from post-SRS BM recurrence.

**Figure 3.**
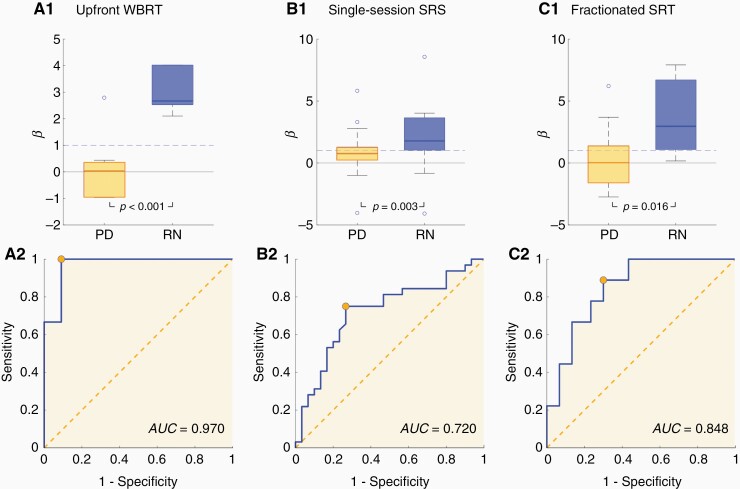
Box plots comparing the growth exponents obtained for recurrence of BM vs radiation necrosis (RN) events and their respective receiver operating characteristic curves for: (A) brain metastases (BMs) with upfront WBRT (PD, *n* = 11; RN, *n* = 9), (B) BMs which received single-session SRS (PD, *n* = 30; RN, *n* = 32) and (C) BMs treated with fractionated SRT (PD, *n* = 30; RN, *n* = 9).

Consequently, an analysis to discriminate between PD and RN similar analysis was performed for the following 3 subgroups: (1) The subset of BMs treated with WBRT before either FSRT or SRS (*n* = 20) (Figure 3A) showed the best distinction with *P < .001* and AUC = 0.970, with 1.000 sensitivity and 0.909 specificities., (2) The subgroup treated with single-session SRS (*n* = 62) (Figure 3B) presented a *P*-value of *P = *.003 and AUC = 0.720, with 0.750 sensitivity and 0.733 specificities., and (3) The last subgroup, BMs treated with fractionated SRT (*n* = 39) (Figure 3C) displayed *P = .016* and AUC = 0.848, with 0.889 sensitivity and 0.700 specificities.

It was further studied whether the rate of volumetric increase obtained from only 2 consecutive magnetic resonance images was able to discriminate between RNs and PDs. The analysis was performed as described in “Methods.” Both comparisons, ie, using the first pair of data points (*V*_*0*_*, V*_*1*_; *t*_*0*_*, t*_*1*_) to obtain $\lambda_{1}\;$, and then using  (*V*_1_*, V*_2_; *t*_1_*, t*_2_) to obtain $\lambda_{2},\;$showed statistically significant differences (*P* = .012 and *P* = .009). When the first 2 points were considered, PD gave higher values for the growth rate (ie, $\lambda_{1}\left(PD\right)\;>\;\lambda_{1}\left(RN\right)\;$), while for the last 2 points, the growth rates were found to be larger for RN events (ie, $\lambda_{2}\left(PD\right)\;<\;\lambda_{2}\left(RN\right)\;$). Figure 2D shows the box plots for the 2 groups. Thus, the exponent was dependent on the time of the measurement and hence was not able to provide a reliable metric to distinguish between these 2 types of response to SRS.

In general, BMs are small, and no limit in size was considered for our study. To study whether small variations in measured volumes due to the segmentation or acquisition procedures could affect the value of the computed growth exponent *β*, a sensitivity analysis was performed as described in “Methods-Sensitivity analysis.” It was found that some lesions' growth exponents were indeed sensitive to variations up to ±5% in volumes. Between 72% and 80% of our dataset was found to be robust to small volume variations. A complementary analysis was performed including only robust BMs. When using the average value on several repetitions and adding random variations, the obtained *P*-value for the Kruskal-Wallis test was *P* = .002 and for the receiver operating characteristic curve, *AUC* = 0.723 (Supplementary Figure S1). When using the median, *p* = 0.001 and *AUC* = 0.718 were obtained (Supplementary Figure S2). The results were consistent with either choosing the average or the median value to represent the small variability for the exponent β.

## Discussion

Patients with BMs often display an increase in the lesion volume as measured in T1-weighted MR imaging after FSRT/SRS, which leads to the differential diagnosis of RN vs PD. The crucial finding was that these 2 conditions could be distinguished using their growth dynamics as assessed by a simple mathematical model using 3 consecutive MRI studies.

There is a lack of consensus or guidelines regarding management of progressively enhanced BMs after FSRT/SRS. Different discrimination methods have been proposed in the literature based on either perfusion MR imaging,^8^ positron emission tomography,^9^ the use of AI,^10^ radiomics,^11,12^ or changes in the shape or anatomical boundaries.^13^ However, many of those methods have not been validated or require complex computational methodologies. Moreover, many of the AI-based studies require extensive validation before clinical implementation due to the limitations of using these multivariate methods on small patient datasets. Mathematical growth models have been shown to provide relevant information in different contexts in (neuro-)oncology,^14,19^ but they are not yet in use in the clinical setting.

In contrast to radiomic studies, where features are learned from patterns in unlabeled datasets and a validation dataset is needed to measure the performance of the learning algorithm,^20^ this study was designed to validate a single hypothesis, whether growth laws could be able to discriminate recurrent BMs from RN events. It was found that when 3 consecutive MRI studies were available, computing the growth exponent *β* could allow lesions to be classified using their growth dynamics as either RN or BM recurrence.

In our dataset, most imaging studies were performed every 3 months, as recommended. Our study raises the question of whether the standard monitoring interval between magnetic resonance images might be suboptimal for BM patients with the controlled extracranial disease. Undeniably, our study suggests that in growing lesions, reducing follow-up to 2 months using standard MRI studies including the 3D post-contrast T1-weighted sequence would increase diagnostic confidence. 3 MRI studies allowing the growth exponent to be computed would be available in the same time period of six months in which now only 2 MRI studies are available. Our metric has the ability to complement other evidence in differentiating RN from post-SRS BM recurrence and could be implemented in AI classifiers incorporating other variables found to be relevant for RN vs PD discrimination.

It is interesting that RN was seen to show accelerated growth dynamics with larger growth exponents than PD, but not necessarily faster growth rates, as our analysis also shows. Recent mathematical studies based on different types of models have shown that inflammatory processes lead to very fast growth dynamics, faster indeed than BM growth itself, fully in line with our observations reported here.

There is little knowledge of increasing volume of BM post-FSRT/SRS. In a study performed by Patel et al.,^21^ a large cohort of 516 responsive BMs showed one-third of treated lesions increased in size during follow-up. Most lesions enlarge 3–6 months post-FSRT/SRS. However, this enlargement can start as early as 6 weeks post-SRS and may not reach peak volume until 15 months post-SRS.^21^ Previous studies have proposed that the observation of volumetric growth over 2 or more sequential follow-up images after SRS took more than 3 months apart could be a signature of tumor recurrence.^22^ In a recent survey, 69.2% of neurosurgeons, agreed that growth after 2 scans repeated within 45–90 days of each other, is sufficient to identify irreversible radiographic progression on surveillance imaging after SRS for BM.^22^ Our results challenge common neuro-oncological beliefs.

However, all the RN events satisfied this condition and were obviously not recurrences. Indeed, our analysis showed that growth rates were unable to distinguish between the 2 conditions and led to conflicting results depending on the timing of the imaging study. There are very few studies regarding volumetric trends in BMs after radiotherapy. Putz et al. showed that the largest volumetric increases compared to baseline corresponded to RN (up to 800%), in consecutive imaging, but no kinetics or model was made.^19^ Moreover, the common definition that lesions that satisfied the Response Assessment in Neuro-Oncology for BMs (RANO-BM) volumetric criteria for progression but showed spontaneous regression during subsequent imaging follow-up should be classified as RN instead of progression should be reconsidered.^23^ The study shows that RN grows more steeply than PD in clinical practice.

BMs treated with fractionated SRT are slightly better classified as PD or RN (AUC = 0.848) than the ones treated with single-session SRS (AUC = 0.720). However, it is also interesting that for the same number of PD in each treatment group (*n* = 30), single-session SRS gave place to 32 RN, while fractionated SRT group only had 9 RN events. In the less common case of BMs with upfront WBRT, the classification was the best of all the studied groups (AUC = 0.970).

The practical implementation of the method developed here requires the volumetric calculation of the size of the lesion. With the advent of many AI packages able to perform automatic segmentation of BMs this calculation should not impose a substantial burden on the radiologist. Time required for segmentation ranges from around 2 minutes for small BMs (< 1 cm^3^) to 15 minutes for the largest ones (> 10 cm^3^). The estimation of *β* requires the segmentation of 3 different time points. Although this process could seem to be time-consuming in some cases and the discriminatory power of our results is moderate for single-session patients, performing the proposed analysis may provide additional relevant information to guide therapeutic decisions. Also, fitting the data to the Von Bertalanffy growth law is necessary to obtain the growth exponent *β*, but this is a mathematically very simple task. It would be easy to develop online calculators providing both the growth exponent and a measure of how likely it is that the observed exponent corresponds to either a RN or a PD.

A limitation of our study was the sample size. After evaluating 1133 BMs, 101 lesions met the inclusion criteria. The inclusion criterion limiting the use of many BMs was the need to have 3 consecutive imaging studies with an increase in tumor volume. On the one hand, if there is no volume increase, no distinction between RN and recurrence would be needed. On the other hand, 3-time points (with an increase in volume) are needed to be able to obtain the growth exponent using the Von Bertalanffy equation. So, unfortunately, less strict criteria cannot be used in this study. Other limitations were the retrospective nature of our study and the fact that different systemic therapies were used. It had been thought that concurrent immunotherapy could increase the likelihood of RN, but recent evidence has refuted this perception.^24^ The strengths of this study were the use of data from several institutions, the use of standard MRI imaging modalities, and the quality of the semi-automatic segmentation, performed by the same expert and confirmed by a radiologist, ensuring the accuracy of the computed volumes used for the study.

In conclusion, we have shown that using 3 consecutive imaging follow-ups, growth dynamics provide a tool to distinguish recurrent BMs from RN. Thus, the growth exponent could help physicians to take a decision together with other variables and thus avoid the use of more invasive methods such as brain biopsy or surgery.

## Supplementary Material

vdac179_suppl_Supplementary_Material
