# Supplementary material for: Growth dynamics of brain metastases differentiate radiation necrosis from recurrence
Source: Neurooncol Adv. 2022 Dec 8;5(1):vdac179. doi: 10.1093/noajnl/vdac179 (PMC9887079; doi:10.1093/noajnl/vdac179)
Supplement: vdac179_suppl_Supplementary_Material [file vdac179_suppl_supplementary_material.pdf]

Neuro-Oncology Advances

Supplementary material for

"Growth dynamics of brain metastasis differentiate radiation necrosis from recurrence"

Beatriz Ocaña-Tienda[1]*, Julián Pérez-Beteta[1], David Molina-García[1], Beatriz Asenjo[2], Ana Ortiz de Mendivil[3], David Albillo[4], Luís A. Pérez-Romasanta[5], Elisabeth González del Portillo[5], Manuel Llorente[4], Natalia Carballo[4], Estanislao Arana[6], Víctor M. Pérez-García[1]

1 Mathematical Oncology Laboratory, University of Castilla-La Mancha, Ciudad Real, Spain.
2 Department of Radiology, Hospital Regional Universitario Carlos Haya, Málaga, Spain.
3 Department of Radiology, Sanchinarro University Hospital, HM Hospitales, Madrid, Spain.
4 Radiology Unit, MD Anderson Cancer Center, Madrid, Spain.
5 Radiation Oncology Service, Salamanca University Hospital, Salamanca, Spain.
6 Department of Radiology, Fundación Instituto Valenciano de Oncología, Valencia, Spain.

***Corresponding Author: Beatriz.Ocana@uclm.es**

Supplementary Figures

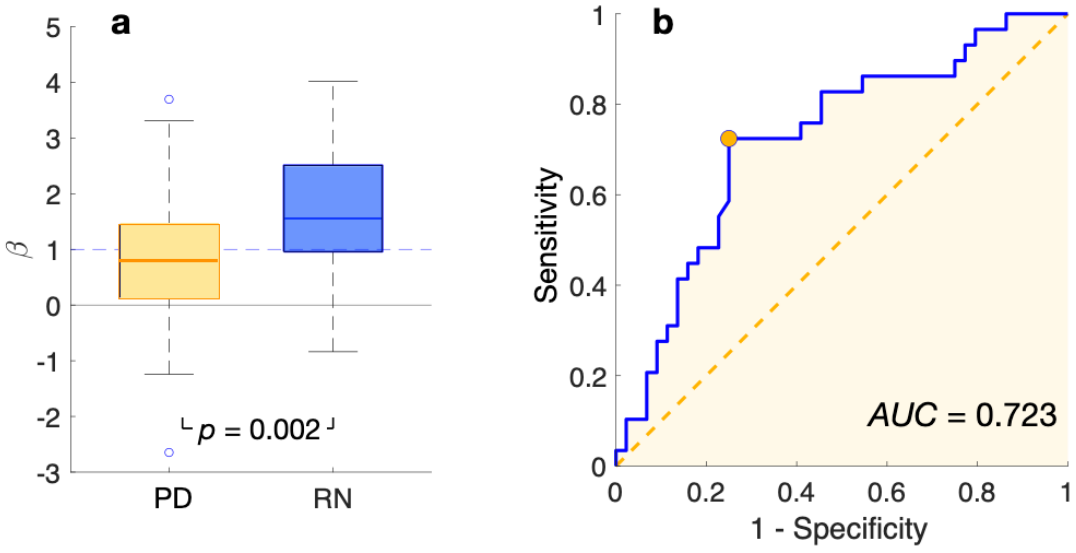

Figure S1. Results after sensitivity analysis when using the average of the 200 computed β*. **a.** Box plots comparing the growth exponents β obtained for recurrence post-SRS of BMs (PD, n=44) versus RN events (n=29). **b.** ROC curve for the discrimination between relapsing BMs and RN.

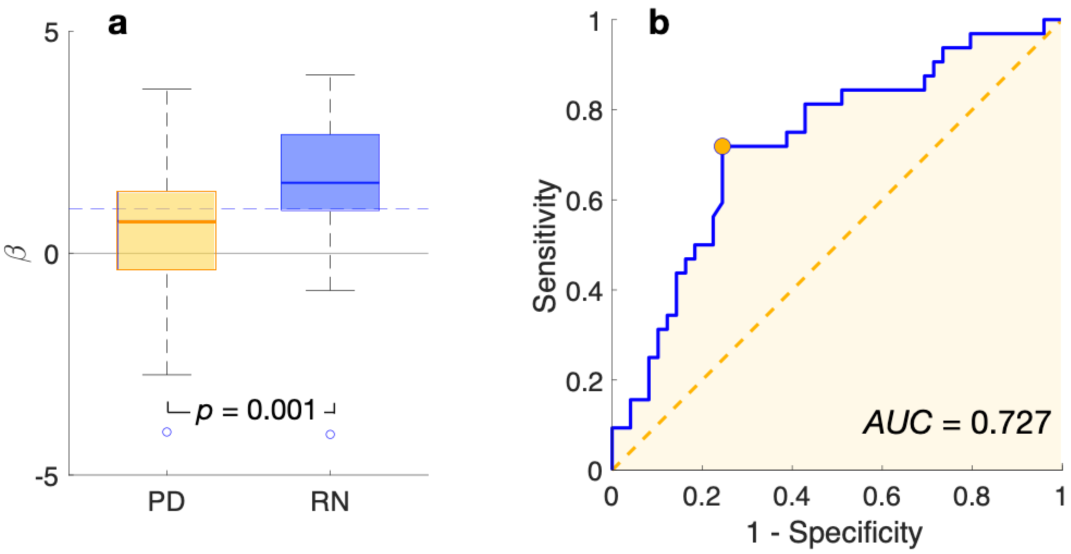

Figure S2. Results after sensitivity analysis when using the median of the 200 computed β*. **a.** Box plots comparing the growth exponents β obtained for recurrence post-SRS of BMs (PD, n=44) versus RN events (n=29). **b.** ROC curve for the discrimination between relapsing BMs and RN.

Supplementary Table

Chemotherapeutic drugs type	Patients n (%)
Erlotinib	6 (14%)
Pemetrexed	6 (14%)
Afatinib	3 (7%)
Nivolumab	3 (7%)
Bevacizumab	2 (5%)
Capecitabine + Lapatinib	2 (5%)
Vinorelbine + carboplatin	2 (5%)
Ado-trastuzumab emtansine (T-DM1)	1 (2%)
Carboplatin + Atezolizumab + Etoposide	1 (2%)
Carboplatin + Atezolizumab + Paclitaxel	1 (2%)
CDDP-VP16	1 (2%)
Docetaxel	1 (2%)
Gemcitabine	1 (2%)
Lorlatinib	1 (2%)
Osimertinib	1 (2%)
Paclitaxel	1 (2%)
Pembrolizumab	1 (2%)
Pemetrexed + Platinum	1 (2%)
Sorafenib	1 (2%)
Sunitinib	1 (2%)
Trastuzumab + Pertuzumab	1 (2%)
Trastuzumab + Vinorelbine	1 (2%)
Trastuzumab + Lapatinib	1 (2%)
Vemurafenib	1 (2%)
Vinorelbine	1 (2%)

Table S1. Chemotherapeutic drugs received by the patients in study
